# Response: Commentary: Using Virtual Reality to Assess Ethical Decisions in Road Traffic Scenarios: Applicability of Value-of-Life-Based Models and Influences of Time Pressure

**DOI:** 10.3389/fnbeh.2018.00128

**Published:** 2018-06-26

**Authors:** Leon R. Sütfeld, Richard Gast, Peter König, Gordon Pipa

**Affiliations:** ^1^Institute of Cognitive Science, Osnabrück University, Osnabrück, Germany; ^2^Nuclear Magnetic Resonance Unit, Max-Planck-Institut für Kognitions- und Neurowissenschaften, Leipzig, Germany

**Keywords:** moral decision making, virtual reality, self-driving cars, moral judgment, ethical decisions, behavioral assessment

In the paper discussed (Sütfeld et al., 2017), we examined the feasibility of using virtual reality (VR) as an assessment method for models of human moral behavior in road traffic scenarios. Furthermore, this experimental approach allowed us to analyze the applicability of logistic regression-based value-of-life models for modeling human behavior. We consider this study to be a contribution to the discussion about ethical decision-making systems in autonomous vehicles (AVs).

## The chosen assessment approach

In a recent commentary on this paper, Keeling (2017) brings up two objections to the approach:

- In the initial study, we cite evidence showing that human moral intuitions differ depending on a variety of contextual variables. Keeling argues that we use this to infer the validity of the meta-ethical position of particularism (Dancy, 1983), and that this inference is not necessarily justified. He further contends that our “answer to the moral design problem depends on the plausibility of this inference.”

In our paper, we argue on the level of moral behavior and moral intuitions, which we can experimentally assess and describe. From the evidence cited, we conclude that these are indeed highly dependent on contextual factors. We thus argue that in order to learn about our behavior and moral intuitions in a particular real-world scenario, it is reasonable to match the contextual factors of the assessment with those of the scenario in question, making the case for a VR assessment as a starting point for this line of research. The experimental data presented and the conclusions based on it are, therefore, not dependent on a specific position in the views on particularism vs. generalism, but independent of this controversy.

- Keeling argues that we are committed to the claim that “the right thing to do in AV collisions is determined by facts about human snap-judgements,” and that this is not a valid claim, since “[h]umans are sensitive to the pressures of a collision, and under this pressure, our critical thinking capacities break-down.”

The used term “snap-judgements” refers to the severe time constraints we encounter in real-life situations of this kind, and it implies that the decisions are likely to differ qualitatively from more elaborated decisions. However, the study used two conditions differing in the degree of time pressure. They delivered qualitatively similar results, giving us no indication that the cognitive processes leading to decisions in such situations differ qualitatively within the time range investigated. Furthermore, for the longer of the two conditions (4s), we observe a surprisingly high amount of consistency, which is at odds with the idea of a “break-down of critical thinking capacities”. If, or to what extent even longer decision time frames might impact the decision outcome remains to be investigated, but we can take the high consensus among the participants as an indicator that the decisions made were far from arbitrary.

Beyond this, we see time constraint as only one of several factors in the assessment methodology that can potentially play a role in our decisions. These factors, including the level of abstraction (e.g., text-based vs. naturalistic), and the level of immersion in the presentation (e.g., immersive VR vs. desktop VR), should be experimentally investigated to get a clearer picture of how stable our moral intuitions are across assessment modes, and to what extent the decision patterns in one assessment mode may give a valid and generalizable account of our moral intuitions for the setting in question.

## Applicability of the modeling approach

With respect to what *ought* to be done and the real-world applicability of the approach suggested, we need to make a distinction between the empirical findings we report and the general approach used to model moral decisions.

First, probabilistic decision-making systems seem unavoidable for self-driving cars. Situations encountered in real life are almost never prototypical, and there is a large number of factors that can play a role in our moral assessment. To what degree and with which probability each of a large number of factors is present in a given situation varies on a continuous spectrum. Any categorical decision-making system will thus either fail to capture all possible combinations of circumstances, have arbitrary decision boundaries, or it will be too large to be fully comprehensible.

Second, we showed that value-of-life-based logistic regression models are generally suited to describe the empirically observed decisions of our subjects in this kind of dilemma situation. Using this class of models, however, does not mean we suggest to unreflectively copy human behavior for cars. Since the straightforward interpretability of the model parameters allows us to superimpose higher-level rules, initial model parameters, possibly obtained by an empirical assessment, could be modified, and isolated factors could be excluded from the model to make it comply with existing jurisdiction or normative theories.

## Applicability of empirical observations

The question remains as to whether ethical decision-making systems based on the empirical assessment of moral intuitions are preferable to systems based on more elaborate and/or normative approaches. In this context, Keeling suggests reverting to “one of our best moral theories, such as utilitarianism or contractualism.” However, describing a moral theory as “best” places it on a quantitative scale, questioning the normative character of the analysis. By proposing two competing moral theories, Keeling also brings up the issue that to this date, no agreement has been reached about which of the various moral theories is the right one. Moreover, normative theories typically come with considerable shortcomings for the purpose at hand. Utilitarianism, for instance, would suggest the sacrifice of innocent bystanders if it means reducing the overall harm. It would further propose colliding with the best protected opposing party (potentially punishing cyclists for wearing a helmet), and it lacks objective quantification of harm for the different options in a given situation. The latter point is particularly problematic, as it leaves us without concrete guidelines on how to behave in more complex situations. Therefore, we cannot *simply* revert to one of the established normative theories. Empirical observations, on the other hand, can deliver a frame of reference for higher-order considerations:

- They can guide the decision between different normative theories in situations where these contradict each other.
- They can deliver initial (numerical) values for mathematical models used in AVs, i.e., they can guide the quantification of rules where the qualitative direction is provided by higher-order rules.
- They can highlight aspects that are underrepresented in normative moral theories, but may play a decisive role in our behavior (e.g., the value of animals).
- They can highlight where normative theories may be at odds with the moral intuitions in a society, and can thus motivate a re-evaluation of particular aspects of a particular normative theory.

In conclusion, we argue that empirical observations play an important role in informing the debate, as well as in determining the rules for implementing moral decision-making systems for AVs.

## Author contributions

LS: main author, wrote most of the manuscript; RG: co-author, helped to improve the manuscript, PK, GP: senior authors, helped to improve the manuscript.

### Conflict of interest statement

The authors declare that the research was conducted in the absence of any commercial or financial relationships that could be construed as a potential conflict of interest.
